# Magneto-Dendrite Effect: Copper Electrodeposition under High Magnetic Field

**DOI:** 10.1038/srep45511

**Published:** 2017-04-04

**Authors:** Makoto Miura, Yoshinobu Oshikiri, Atsushi Sugiyama, Ryoichi Morimoto, Iwao Mogi, Miki Miura, Satoshi Takagi, Yusuke Yamauchi, Ryoichi Aogaki

**Affiliations:** 1 Hokkaido Polytechnic College, Otaru, Hokkaido 047-0292, Japan; 2 Yamagata College of Industry and Technology, Matsuei, Yamagata 990-2473, Japan; 3 Yoshino Denka Kogyo, Inc., Yoshikawa, Saitama 342-0008, Japan; 4 Research Organization for Nano and Life Innovation, Waseda University, Shinjuku, Tokyo 162-0041, Japan; 5 National Institute for Materials Science, Tsukuba, Ibaraki 305-0044, Japan; 6 Saitama Prefectural Showa Water Filtration Plant, Kasukabe, Saitama 344-0113, Japan; 7 Institute for Materials Research, Tohoku University, Aoba-ku, Sendai 980-8577, Japan; 8 Yokohama Harbor Polytechnic College, Naka, Yokohama 231-0811, Japan; 9 Koriyama Technical Academy, Koriyama, Fukushima 963-8816, Japan; 10 Polytechnic University, Sumida, Tokyo 130-0026, Japan

## Abstract

Ionic vacancy is a by-product in electrochemical reaction, composed of polarized free space of the order of 0.1 nm with a 1 s lifetime, and playing key roles in nano-electrochemical processes. However, its chemical nature has not yet been clarified. In copper electrodeposition under a high magnetic field of 15 T, using a new electrode system called cyclotron magnetohydrodynamic (MHD) electrode (CMHDE) composed of a pair of concentric cylindrical electrodes, we have found an extraordinary dendritic growth with a drastic positive potential shift from hydrogen-gas evolution potential. Dendritic deposition is characterized by the co-deposition of hydrogen molecule, but such a positive potential shift makes hydrogen-gas evolution impossible. However, in the high magnetic field, instead of flat deposit, remarkable dendritic growth emerged. By examining the chemical nature of ionic vacancy, it was concluded that ionic vacancy works on the dendrite formation with the extraordinary potential shift.

Electrodeposition plays a major role for the fabrication of electrocatalysts, which are expected to make great contribution to future energy conversion and storage technologies such as water electrolysers and fuel cells. These applications are potentially useful as power sources in transportation by vehicles emitting carbon dioxide and consuming fossil fuels. Other aspects of catalyst such as stereoselectivity and chirality are also issues of paramount importance. Chirality is a fundamental concept in chemistry and life science, and chiral catalysts play the most important scientific and technological roles in modern industry, especially in pharmaceutical sectors. In this sense, how to fabricate chiral catalysts is still an open question with important fundamental and technical interest. Magnetic field provides a powerful tool of fabrication for a new type of chiral electrocatalyst; by means of magnetic field and macroscopic rotation, numerous chiral screw dislocations are formed on electrode surfaces, which can donate various chiral activities to crystal nuclei^1^,^2^,^3^,^4^. These catalytic activities appear on flat surfaces. However, regarding morphological aspect, a different type of crystal growth exists, i.e., dendritic crystallization, which is composed of three-dimensionally ramified nuclei, continuously branching off from substrate.

In electrodeposition, dendritic growth is often observed together with hydrogen-gas evolution^5^,^6^,^7^,^8^,^9^,^10^. Dendrite formation is therefore quite popular in zinc deposition^8^,^9^,^10^, of which reduction potential is much more cathodic than the normal hydrogen potential (NHE). Even in copper deposition whose equilibrium potential is more anodic than NHE, if the electrode potential is shifted to much more cathodic side than NHE, dendrite easily emerges with hydrogen evolution^5^,^6^,^7^.

However, a quite different type of dendritic growth without hydrogen evolution has been recently found in copper deposition by using a new type of electrode system called cyclotron magnetohydrodynamic (MHD) electrode (CMHDE). Figure 1A represents the current-potential curves for copper cathodic deposition from acidic copper sulfate solution in the presence (a) and absence (b) of magnetic field. In zero magnetic field, electrolytic current steeply rises at −550 mV (the hydrogen evolution potential, *E*_H2_ vs. NHE) with hydrogen-gas evolution, whereas at a 15 T magnetic field, the current-rising potential is drastically shifted to anodic side by 360 mV (at −190 mV vs. NHE) from the original hydrogen evolution potential, which implies that the rising current is unrelated to hydrogen-gas evolution. Since the increment of potential energy by a 15 T magnetic field is estimated only a few milli-eV, such a potential shift cannot be explained by any conventional theoretical basis. Actually, for copper magnetoelectrodeposition in an ordinary electrolysis cell such as cells with wire and hanging electrodes, we have not observed such a potential shift. Figure 1B exhibits the SEM images of the corresponding deposit surfaces; though no hydrogen-gas evolution, in the high magnetic field, remarkable copper dendritic growth is observed [Fig. 1B(a)], which is quite similar to the ordinary dendrite by the co-deposition of hydrogen molecules in zero magnetic field [Fig. 1B(b)]. This means that in place of hydrogen, something else works on copper nucleation, promoting the dendritic growth with rising deposition current.

In recent years, under high magnetic fields, microbubbles without electrochemical gas-evolution of hydrogen or oxygen has been continuously found in ferricyanide-ferrocyanide redox reaction^11^, copper cathodic deposition^12^ and copper anodic dissolution^13^. As will be elucidated later, such experimental results are attributed to the production of ionic vacancy in electrode reactions.

Ionic vacancy is a polarized free space of the order of 0.1 nm surrounded by oppositely charged ionic cloud, which has been known as one of point defects in solid electrolyte^14^,^15^,^16^,^17^. However, in the present case, ionic vacancy is surrounded by quite different environment, i.e., aqueous electrolyte. Electron transfer producing ionic vacancy takes place in a quite short period of the order of 10^−15^ s, and even in the case of stabilized hydrated electron, the lifetime of the electron is estimated at most of the order of 10^−12^ s^18^,^19^,^20^. Therefore, it has been generally recognized that if evolved, the vacancy would be instantaneously annihilated. However, from the measurement by CMHDE, it was clarified that the lifetime is extremely long, i.e., 1 s^21^, which is long enough that ionic vacancies are transformed to nanobubbles^22^. Then, the nanobubbles are coalesced into microbubbles. At the same time, it was concluded that by using CMHDE, the concentration of ionic vacancy on the electrodes can be controlled by magnetic field, i.e., CMHDE can provide various vacancy concentrations to electrode reactions. The experimental results in Fig. 1A,B were actually obtained by the CMHDE under zero and 15 T magnetic fields.

The purpose of the present paper is by means of the characteristic feature of CMHDE to experimentally make clear the mechanism of the magneto-dendrite effect shown in Fig. 1A,B, i.e., the extraordinary dendritic growth without hydrogen-gas evolution accelerated by the adsorption of ionic vacancy under the control of magnetic field.

## Results and Discussion

Nanobubbles have been recently paid much attention as the smallest bubbles^23^,^24^,^25^,^26^,^27^,^28^,^29^,^30^. However, ionic vacancy provides them in quite different way. Figure 2A exhibits the microbubbles observed in copper electrodeposition under an 8 T vertical magnetic field. Universality of the phenomenon^11^,^12^,^13^ allowed us to conclude that via. nanobubbels, the observed microbubbles arise from ionic vacancies created by electrode reactions. As shown in Fig. 2B, in the electrolysis under a vertical magnetic field, a tornado-like rotation called vertical MHD flow emerges over electrode surface, of which radial secondary flow gives rise to a collision field for created ionic vacancies at the electrode center, where ionic vacancies collide to yield nanobubbles of the order of 1 nm containing dissolved gas^22^, and the created nanobubbles are in turn converted to observable microbubbles via. Ostwald ripening^11^,^12^,^13^.

Figure 3A shows the schematic structure of an ionic vacancy in cathodic reaction; negative inner surface arises from polarized water molecule^31^. Therefore, we can expect that ionic vacancy has the chemical nature of specific adsorption shown by hydroxide ion or proton. At the same time, the process of vacancy formation has been theoretically made clear^31^; the exact equation of motion based on Newton’s second law of motion demands the conservation of momentum at electron transfer in electrode reaction. On the other hand, in accordance with Frank-Condon principle, electronic transfer from or to electrode is so fast that it can be regarded as taking in a stationary nuclear framework, so that the momentums of reactant and activated complex are equalized to zero. This implies that the momentum of transferring electrons must be compensated by the emission of initial embryo vacancy to solution side. Frank-Condon principle also requires the conservation of electric charge; since the reaction system is regarded as forming a stationary nuclear framework, the change in the electric field between the initial and activated states of reaction can be neglected. As a result, the transferring electrons induce dielectric polarization on the inner wall of the vacancy with the same electric charge. Figure 3B, C shows the emission of ionic vacancy from the collision of electrons to reactant and the polarization of the inner wall in case of cathodic reaction, respectively. The emitted embryo vacancy develops to a steady-state vacancy by the thermal motion of solution particles^32^.

The lifetime of ionic vacancy was measured by a CMHDE, which is, as shown in Fig. 4A(a), composed of a pair of concentric cylindrical electrodes partly shielded [Fig. 4A(b)], where electrolytic current flows between the inner and outer electrodes, so that a parallel magnetic field to the electrode surfaces yields a tangential Lorentz force for the MHD flow circulating along the cylindrical walls. In a low magnetic field, due to low velocity, ionic vacancies created are extinguished on the way of returning, i.e., the MHD flow is kept in viscid mode [Fig. 4A(c)]. However, in a high magnetic field, owing to high velocity, they survive the circulation to cover the whole surfaces, so that the flow turns from viscid to inviscid [Fig. 4A(d)]. In accordance with the theoretical equations^21^, in viscid mode, the diffusion current is proportional to the 1/2nd power of magnetic flux density, whereas in inviscid mode, it is in proportion to the 1st power of magnetic flux density. Detecting the change in the flow mode by diffusion current, we have succeeded to determine the lifetime of ionic vacancy.

**Figure 1:**
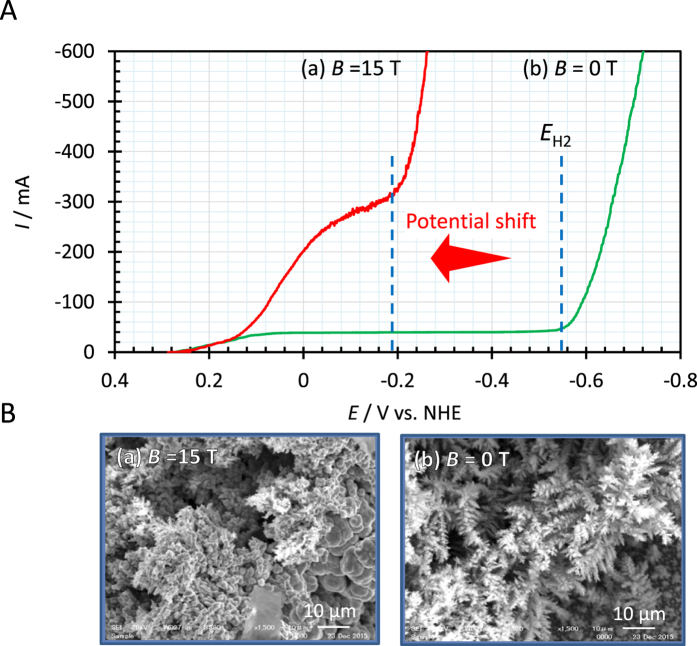
Potential shift of 360 mV in anodic direction observed in copper deposition by cathodic potential sweep at a 15 T magnetic field and SEM images of the corresponding dendritic deposition. (**A**) Current *vs.* potential curves; (a) Red solid line, *B* = 15 T; (b) Green solid line, *B* = 0 T. *E*_H2_, Hydrogen evolution potential; [CuSO_4_], 300 mol m^−3^; [H_2_SO_4_], 500 mol m^−3^; Temperature, 23 °C; Sweep rate, 5 mVs^−1^. Electrode, Copper-CMHDE. (**B**) SEM images of copper dendrites obtained after the potential sweeps. (a) *B* = 15 T without hydrogen gas evolution. (b) *B* = 0 T with hydrogen gas evolution.

**Figure 2:**
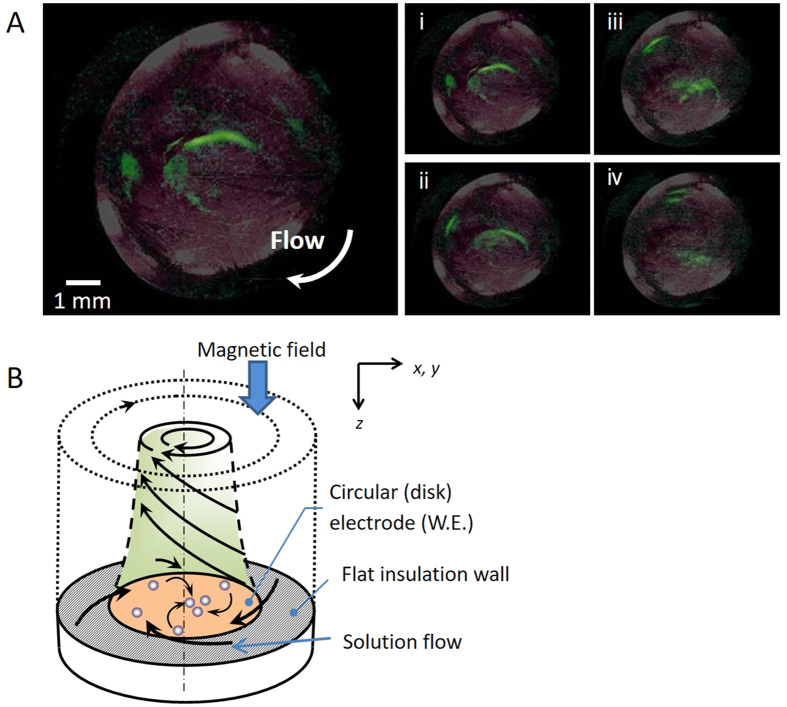
Microbubble formation in copper deposition under a vertical MHD flow. (**A**) Photographs (i) to (iv) taken at the interval of 0.07 s. For convenience, the bubbles are painted green. Experimental conditions; [CuSO_4_] = 30 molm^−3^; [H_2_SO_4_] = 100 molm^−3^. *B* = 8 T, *V* = −144 mV (+125 mV vs. NHE). Potential was swept from the rest potential (+269 mV vs. NHE) to −300 mV (−31 mV vs. NHE) at 1 mVs^−1^. (**B**) Vertical MHD flow over an electrode in a vertical magnetic field and a sample setup into a superconducting magnet.

**Figure 3:**
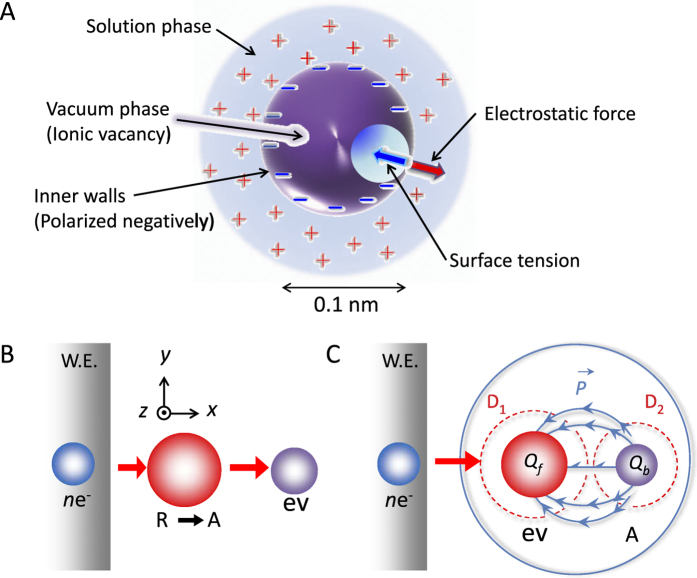
Structure and formation process of ionic vacancy in cathodic reaction. (**A**) Structure of ionic vacancy, (**B**) Conservation of momentum and (**C**) Conservation of electric charge. *n*e^-^, transferring electrons; R, reactant; A, activated complex; ev, initial embryo vacancy; *Q*_*f*_, free electric charge introduced; *Q*_*b*_, polarized electric charge on the inner wall of vacancy; W.E., working electrode. D_1_ and D_2_ are the domains enclosing activated complex and embryo vacancy, respectively.

**Figure 4:**
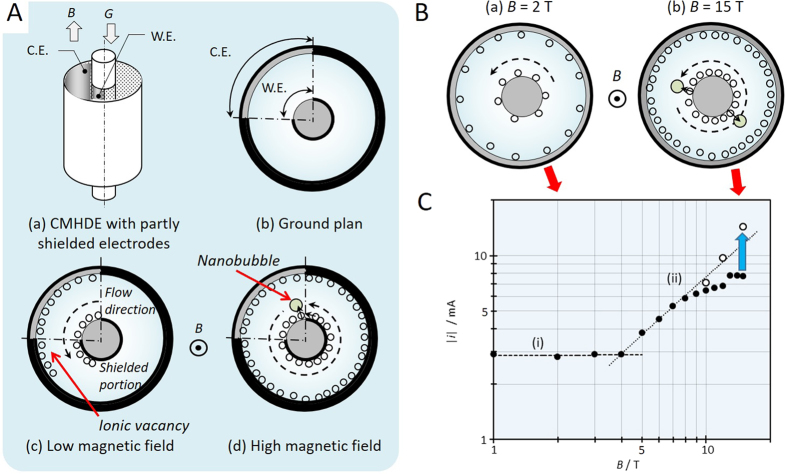
Control of vacancy concentration by CMHDE. (**A**) Measurement of the lifetime of ionic vacancy by CMHDE. The lifetime measured was 1.2 s. (a) Schematic of CMHDE with partly shielded electrodes, (b) Ground plan, (c) Viscid flow in a low magnetic field, and (d) Inviscid flow in a high magnetic field. W. E., working electrode; C. E., counter electrode; *B*, magnetic flux density, *G*, gravity field. (**B**) Saturated and unsaturated vacancy concentrations in completely exposed CMHDE. (a) Unsaturated vacancies at 2 T, and (b) Saturated vacancies at 15 T. (**C**) Current response against magnetic flux density by completely exposed CMHDE. (i) The range of low vacancy concentration, (ii) The range of high vacancy concentration. [H_2_SO_4_] = 100 mol m^−3^; [CuSO_4_] = 30 mol m^−3^; Applied overpotential, *V* = −250 mV (−9 mV vs. NHE). ●, Current in steady state; ○, Current after bursting hydrogen bubbles. The broken lines represent theoretical ones with slopes of 0 and 1.

Figure 1A,B was obtained not by partly shielded CMHDE, but by completely exposed CMHDE under zero and 15 T magnetic fields. As shown in Fig. 4B, magnetic field parallel to the completely exposed CMHDE also induces a circular flow of electrolyte solution between the completely exposed cylinder walls. In a low magnetic field, due to low circulating velocity, ionic vacancies created become extinct [Fig. 4B(a)], whereas in a high magnetic field, owing to high velocity, they can survive, and cover the wall surfaces [Fig. 4B(b)]. Differently from the case of partly shielded CMHDE shown in Fig. 4A, in the present case, due to the rotational symmetry of the electrode, the diffusion layer in viscous laminar flow under a low magnetic field can not develop along the electrode, i.e., independent of magnetic field, so that the diffusion current is kept constant, of which value is determined by natural convection. In a high magnetic field, according to the conversion from viscid to inviscid flow, in the same way as the partly shielded CMHDE, a convective-diffusion layer is formed by induced minute vortexes called micro MHD flow^21^, so that the current depends on magnetic field, therefore, proportional to the 1st power of magnetic flux density. In the viscid mode, due to extinction, ionic vacancy remains in low concentration, whereas in the inviscid mode, it would easily reach saturated state. As shown in Fig. 4B, copper deposition at 2 T is performed in low ionic vacancy concentration [Fig. 4B(a)], and at 15 T, copper atoms are deposited under saturated concentration of ionic vacancy [Fig. 4B(b)]. Figure 4C exhibits a log-log plot of the diffusion-limiting current of copper deposition against magnetic flux density by means of a completely exposed CMHDE. In the region of the constant current, ionic vacancies are extinguished on the way of circulation, so that the surface concentration is kept low, whereas in the current region proportional to the 1st power of magnetic flux density, the cylindrical walls are covered with ionic vacancies, so that the surface concentration of ionic vacancy is high, increasing with magnetic field.

After saturation, collision between ionic vacancies, as shown in Fig. 4B(b), yields nanobubbles adsorbing on the electrode surfaces to block reaction. As a result, in Fig. 4C, the current values of solid circles deviate downward from the plot of a slope 1. To remove the adsorbed nanobubbles, imposition of a sufficiently short potential step beyond the hydrogen evolution potential *E*_H2_ is so effective that bursting evolution of hydrogen gas blows them off. In Fig. 4C, the recovered data after the bursting operation are plotted as blank circles. From these experimental results, it is concluded that ionic vacancies on the electrode are unsaturated at 2 T but saturated at 15 T.

**Figure 5:**
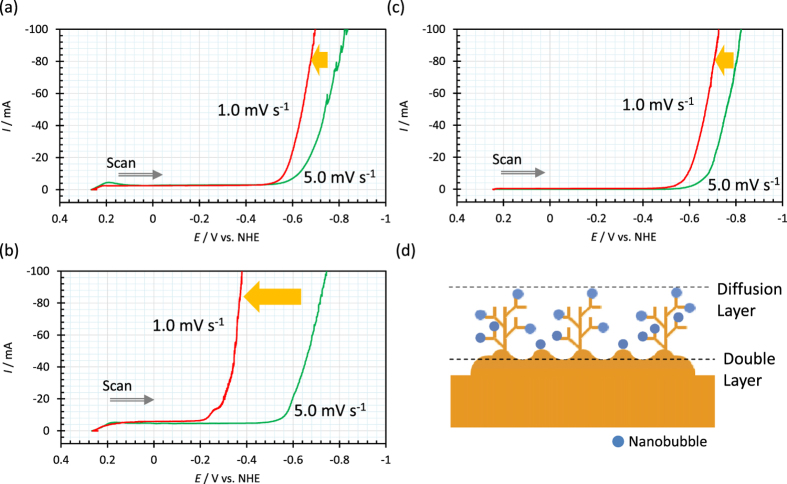
Dependences of current-potential curve in copper CMHDE on sweep rate. (**a**) Copper deposition at 2 T, (**b**) Copper deposition at 15 T, (**c**) Water electrolysis at 15 T. (**d**) Dendrite formation process by the nanobubble adsorption to 3D nuclei. Sweep rate: Red solid lines, 1.0 mV s^−1^; Green solid lines, 5.0 mV s^−1^. The geometrical configuration of CMHDE is the same as shown in Fig. 4B. Solution composition; (**a**) and (**b**) [CuSO_4_] = 30 mol m^−3^ and [H_2_SO_4_] = 100 mol m^−3^; (**c**) [H_2_SO_4_] = 100 mol m^−3^.

To compare current responses against electrode potential at unsaturated and saturated vacancy concentrations in copper deposition, in Fig. 5(a), (b) at magnetic fields of 2 T and 15 T, the electrode potentials were swept in cathodic direction from the rest potential; at 2 T, even if changing the sweep rate, the rising potential is hardly shifted at the same electrode potential as shown in Fig. 1A at 0 T, staying in the vicinity of the hydrogen evolution potential *E*_H2_ [Fig. 5(a)].

However, at 15 T, a quite different result is obtained, i.e., the more the sweep rate is decreased, where the vacancy concentration increased, the more the rising potential is shifted to anodic side apart from the original hydrogen evolution potential [Fig. 5(b)]. This implies that the extraordinary potential shift in Fig. 1A is attributed to the adsorption of saturated ionic vacancy.

Furthermore, to confirm the role of ionic vacancy in electrodeposition, by using the same copper CMHDE, water electrolysis was carried out in 100 mol m^−3^ sulfuric acid solution. Under a magnetic field of 15 T, the electrode potential was swept from the rest potential to cathodic direction. In Fig. 5(c) though the saturated concentration of ionic vacancy is expected, there is no potential shift such as copper deposition shown in Fig. 5(b). Accordingly, it is concluded that ionic vacancy only works for electrodeposition. In general, electrochemical nucleation is classified into two types, i.e., 2D nucleation in electric double layer and 3D nucleation in diffusion layer^33^,^34^,^35^,^36^,^37^. Dendritic growth is induced by hydrogen molecules, which arise from the protons specifically adsorbed on the active points of 3D nuclei, and block their nucleation. Continuous suppression of the active points of nucleation accelerates the branching of nucleus leading to dendritic growth. Figure 5(d) exhibits that nanobubbles from ionic vacancies play the same role as hydrogen molecules on dendritic growth. In this case, differently from the ordinary case of hydrogen evolution, though vacancy production itself does not bring current increase, the branching of 3D nuclei in diffusion layer leads to abruptly rising current.

**Figure 6:**
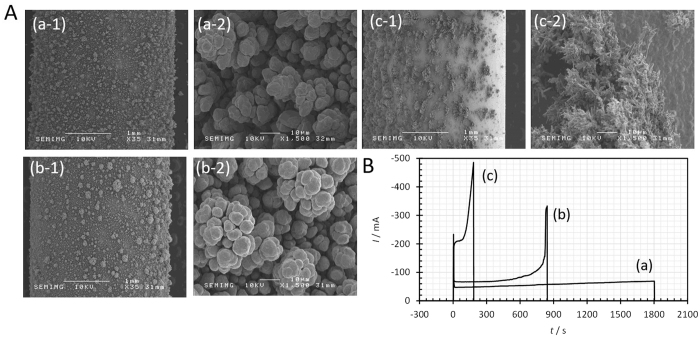
Copper surfaces deposited at a constant potential far away from hydrogen evolution potential by CMHDE and their current responses. (**A**) Deposit surfaces. (a) *B* = 0 T, for 1800 s, (b) *B* = 2 T, for 840 s, (**c**) *B* = 15 T, for 180 s. Applied overpotential *V* = −300 mV (−59 mV vs. NHE). [CuSO_4_] = 300 mol m^−3^; [H_2_SO_4_] = 500 mol m^−3^. Other experimental conditions are the same as shown in Fig. 1B. (**B**) Current-time curves corresponding to (**A**). (a) *B* = 0 T, (b) *B* = 2 T, and (c) *B* = 15 T.

Finally, to ascertain above discussion, another type of copper deposition in the absence of hydrogen-gas evolution was examined, i.e., copper deposition at a constant potential much anodic than the hydrogen evolution potential *E*_H2_. Figure 6A shows SEM images taken at 0 T, 2 T and 15 T. In Fig. 6B, the corresponding current response vs. time curves are represented; at 0 T, larger nodules of coalesced 3D nuclei are observed among smaller nodules after a constant current for 1800 s [Fig. 6A(a)]. At 2 T, nodules of the same type are formed among small ones with an abrupt current rise after 840 s [Fig. 6A(b)]. However, at 15T, different morphology of clusters, i.e., dendrite clusters develop from flat surfaces with a more abrupt current rise after 180 s [Fig. 6A(c)]. These results also suggest that the vacancy concentration controls the morphology of deposit; at a low vacancy concentration in a stationary solution at 0 T or in a viscid flow at 2 T, 3D nucleus nodules are randomly developed, whereas at saturated concentration in an inviscid flow at 15 T, dendrite clusters grow rather in good order. The morphologies of their substrates are also different; the formers are filled with small nodules of 3D nucleus, and the latter is composed of flat surfaces.

In conclusion, as a specifically adsorptive ion, ionic vacancy created in electrode reaction plays the same role as proton in dendritic growth. After receiving electrons, adsorbed protons are changed into hydrogen molecules on the active points of nucleation, leading to typical dendritic growth. In the same way, ionic vacancies are specifically adsorbed at the active points, and due to saturation, transformed to nanobubbles. Blocking the nucleation, they tremendously accelerate the branching of 3D nuclei in diffusion layer, so that electrolytic current abruptly increases. In a high magnetic field, CMHDE supplies saturated ionic vacancies to the active points of copper nuclei. As a result, without hydrogen-gas evolution, the drastic potential shift of 360 mV of rising current emerges at 15 T with remarkable dendrite formation. Dendrite formation is an important technique for the production of micro- and nano-particles. However, hydrogen inclusion is a problem. The usage of ionic vacancy may be a quite prospective solution of the problem.

In addition, in the fabrication of chiral electrocatalysts, ionic vacancy also plays a main role. As mentioned initially, under magnetic fields with macroscopic rotation, chiral screw dislocations are created by microscopic chiral vortexes emerging on free surfaces without friction. Due to lubricant nature, ionic vacancy provides the free surface, contributing to the formation of the chiral catalystic activity.

## Methods

Experimental examination was performed by using the CMHDE completely exposed without shield shown in Fig. 4B. The inner copper cylinder with 1.5 mm radius and 15 mm height was used as working electrode, and the outer copper cylinder with 2.7 mm radius and 15 mm height was used as counter electrode. A calomel electrode was used as reference electrode. Copper deposition was performed in a 300 mol m^−3^ CuSO_4_ + 500 mol m^−3^ H_2_SO_4_ solution or a 30 mol m^−3^ CuSO_4_ + 100 mol m^−3^ H_2_SO_4_ solution. For comparison, with the same CMHDE, water electrolysis was carried out in a 100 mol m^−3^ H_2_SO_4_ solution. During experiment, nitrogen gas was introduced to supply the inner gas of nanobubbles. The whole apparatus was settled in the bore space of the 15 T cryocooled superconducting magnet at the High Field Laboratory for Superconducting Materials, Institute for Materials Research, Tohoku University. The solution was kept at the bore temperature between 22 °C to 24 °C.

## Additional Information

**How to cite this article:** Miura, M. *et al*. Magneto-Dendrite Effect: Copper Electrodeposition under High Magnetic Field. *Sci. Rep.* **7**, 45511; doi: 10.1038/srep45511 (2017).

**Publisher's note:** Springer Nature remains neutral with regard to jurisdictional claims in published maps and institutional affiliations.
